# “A New Hope” for Positive Psychology: A Dynamic Systems Reconceptualization of Hope Theory

**DOI:** 10.3389/fpsyg.2022.809053

**Published:** 2022-02-23

**Authors:** Rachel Colla, Paige Williams, Lindsay G. Oades, Jesus Camacho-Morles

**Affiliations:** Centre for Wellbeing Science, Melbourne Graduate School of Education, University of Melbourne, Parkville, VIC, Australia

**Keywords:** systems dynamics, interdisciplinary, hope theory, methodology, meta-theoretical

## Abstract

In this review of the central tenets of hope theory, we examine the meta-theoretical, theoretical, and methodological foundations of the literature base. Our analysis moves from a broad examination of the research landscape in hope theory across disciplines, to a deeper investigation of the empirical literature in university students. This review highlights the significant impact of this body of research in advancing our understanding of aspects of thriving characterized by hope. However, we also evidence several limitations that may impede the advancement of the next wave of growth in this field. To address these limitations, we argue for an interdisciplinary approach to expanding the meta-theoretical, theoretical, and methodological horizons, enabling a more dynamic systems approach to the study of hope. Drawing on the intersection of positive psychology with systems thinking, we describe a methodological approach that enables a deeper examination of the processes and interactions through which hope emerges, using an analysis of the lived experience of young people. It is proposed that this research agenda will bring to life an alternate story about the resourcefulness of our youth through their own voice, enabling us to leverage this in the design of more effective strategies to facilitate hope in the future. This research agenda provides a roadmap that will provide alternative methodologies that address the current limitations in the field of hope research and, importantly, can provide fuel to spur on the acceleration of the next wave of research and practice in the field of positive psychology more broadly.

## Introduction

“A long time ago in a galaxy far, far away”…. With this phrase, George Lucas launched what was to become one of the most successful cinematic epic sagas in recent history. In a thought-provoking choice, Lucas began the series in the middle of a story, launching with the fourth episode, Star Wars: A New Hope, for technical and storytelling reasons. The story launches straight into the action while hinting at untold history. In a parallel universe, Rick Snyder launched a theory of hope that has helped drive the significant growth and impact of the field of positive psychology, but there is a sense that there was more to this story. As hope theory enters its third decade of research, the first trilogy if you like, many storylines have been explored, with the opportunity now for some deeper character development. In this critically appraised topic (CAT) review, we explore the roots of the hope theory story by examining the meta-theoretical, theoretical, and methodological assumptions that underpin the research. As the story develops, we argue that drawing on multi-disciplinary approaches, such as complex dynamic systems, will help tell some of the “untold history” and thus deepen understanding of the interactions that facilitate aspects of thriving characterized by hope.

Early positive psychology researchers set the tone for significant growth and development of the field, influenced by the desire to provide a systematic and rigorous approach to the scientific study of what enables thriving, or optimal development, across various life domains. As a result, much of the meta-theoretical and methodological development of research and practice has been grounded in a dualist positivist epistemology and realist ontological view of the world as “knowable” (Gergen, 1990). This paradigm sees reality as objectively observable, fixed, and generalizable; as such, it transcends context (Ward et al., 2015). However, the unintended consequence of this has led to one of the most persistent criticisms of the field, namely, that there is a dominant focus on the individual that lacks an appreciation of contextual and dynamic influences on thriving (Kern et al., 2020).

This limitation is epitomized in Snyder et al.’s (1991) hope theory. While Snyder’s conceptualization of hope articulated an iterative and dynamic process between agency (goal-directed energy) and pathway thinking (planning to meet goals), the methods through which it was operationalized and measured produced a more linear and acontextual construct in the empirical literature. We argue that the dynamic tenets of hope theory that have been left on the “editing floor” with this technical limitation provide valuable insights into the mechanisms that enable the core capabilities to develop. Moreover, the theory is framed in an individualistic cultural perspective and thus may lack applicability to more collectivist cultures. For example, in a conjoint perspective of agency, goals, and actions are defined interpersonally rather than individually and often reflect individuals’ interdependence and position within social situations (Bernardo, 2010). This is not represented in the current liberal individualist conceptualization of hope theory.

This manuscript reviews the evolution of research on Snyder’s hope theory and the substantial body of evidence linking hope with adaptive functioning. The review reveals several unanswered questions around the mechanisms that facilitate these links that have not been resolved through current methodological approaches. With a meta-analysis demonstrating only small effect sizes, particularly across different contexts (Weis and Speridakos, 2011), the effectiveness of the translation of this body of research to practice can also be questioned. These limitations suggest the need for a deepened understanding of how hope emerges; one that recognizes and addresses the inherent complexities in the emergence of hope and is grounded in the lived experience of different sociocultural contexts.

Scholars have recently recognized the need to expand positive psychology toward more complex understandings of the factors and contexts influencing wellbeing. For example, Kern et al.’s (2020) proposal of systems informed positive psychology (SIPP) and Lomas et al.’s (2020) illumination of the dynamics that can broaden the field toward complexity. In this manuscript, we take up this call for a broadened perspective to one area of positive psychology—hope theory—and in doing so, provide “a new hope” for how these aspirations can be translated into research practice.

### Our Research Agenda

The research agenda we propose aims to address some of these limitations by taking an integrated multi-disciplinary perspective, expanding the current meta-theoretical, theoretical, and methodological approaches that have underpinned the hope research to date. The model we propose expands Snyder’s conceptualization to incorporate an additional interpersonal factor (*WePower*) and intrapersonal factor (*WhyPower*), in addition to existing elements of hope theory, motivation to succeed (*WillPower*), and planning to meet goals (*WayPower*). The interplay between these factors is a crucial focus of this research agenda, enabling a more dynamic systems model of hope to be developed. In the expanded model we propose, hope is conceptualized as an emergent property that cannot be fully understood by breaking the construct down into its constituent parts; instead, it is an energy system derived from the dynamic interplay between the parts.

A systems approach argues that more profound knowledge and meaningful understanding come from constructing whole pictures and examining the interrelatedness of factors rather than examining factors in isolation (Flood, 2010). Therefore, drawing from the intersection of positive psychology with complex systems dynamics, we describe a methodological approach that enables a deeper examination of the processes and interactions that facilitate hope to emerge through an analysis of the lived experience of young people. This mixed-methods approach addresses the call for more qualitative research in exploring optimal functioning, proposed by Hefferon et al. (2017), without compromising the systematic rigor aspirations of the founding scholars of positive psychology.

It is proposed that this research agenda will bring to life an as yet untold story about the resourcefulness of our youth through their own voice, enabling us to leverage this in the design of more effective strategies to facilitate hope in the future. Our purpose is to offer a roadmap that will provide alternative methodologies to address the current limitations in hope research and offer insights that can enhance research and practice in positive psychology more broadly.

## Hope Rising: An Analysis of the Development of Hope Theory

Hope has captured the attention of philosophers, poets, artists, and scholars throughout the ages. In the late 20th Century, numerous social scientists turned their attention to operationalizing hope, with more than 26 theories or definitions generated (Lopez et al., 2003). There is consistency in the core themes underpinning these different theories, namely, that hope is a human strength that enables individuals to draw on resources in their environment to support pathways toward healthy development and achievement. The vast majority of these theories operationally defined hope as a unidimensional construct grounded in a positive expectation that goals can be met (see Callina et al., 2018 for a review of the history of conceptualizations of hope). However, Snyder et al.’s (1991) two-factor model of hope has dominated the psychological literature over the last 30 years and is one of the key theories underpinning the development of positive psychology.

Snyder et al.’s (1991) theory defines hope as a dynamic motivational experience that is interactively derived from two distinct types of cognitive tools in the context of goal achievement–namely, pathways and agency thinking. His theory proposes that hope results from an individuals’ perceived ability to develop numerous and flexible pathways toward their goals, allowing them to identify barriers and strategies to overcome these as they move toward goal achievement (WayPower). It is further fueled by the individuals’ sense of agency in their goal pursuit, defined as goal-directed energy or determination to succeed (WillPower) (Snyder et al., 1991). These two factors are theorized to be positively related yet distinct (Snyder, 1989). That is, one can have a strong sense of agency without necessarily demonstrating successful pathways planning toward their goals. However, the additive, reciprocal relationship between the two factors results in a cumulative positive experience of hope that provides the dynamic motivation to *act*—a key differentiator of hope theory from other related constructs such as optimism and self-efficacy (Snyder, 2002). The theory has expanded from its original proposition of hope as a trait, or disposition consistent across time and situations, to now include evidence of hope as a state or momentary experience (Snyder et al., 1996), as well as being specific to certain life domains (Robinson and Rose, 2010).

**FIGURE 1:**
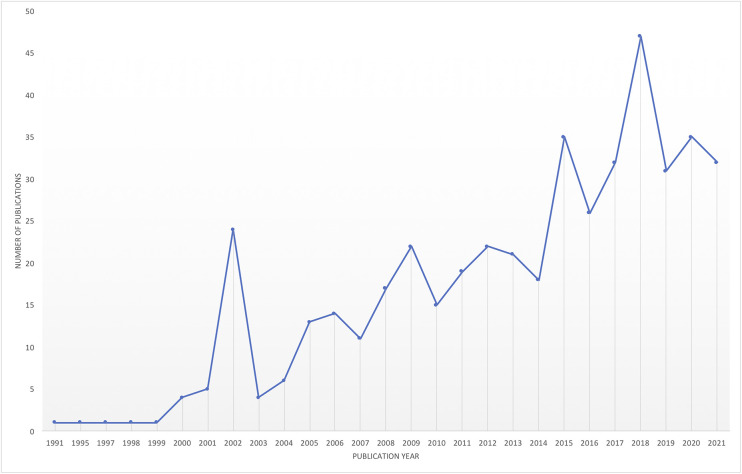
Growth in number of peer-reviewed publications in hope theory by year (1991–2021).

**FIGURE 2:**
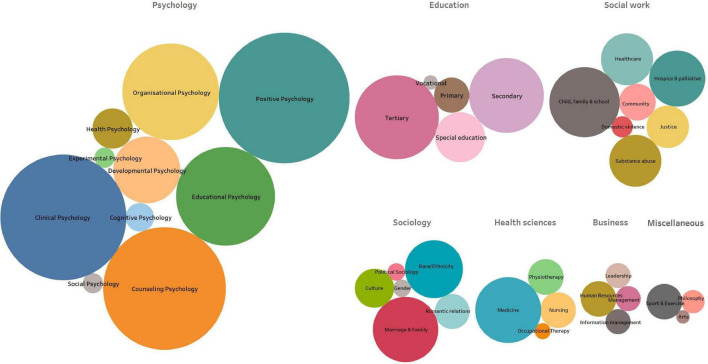
Constellation map showing clusters of application of hope research across disciplines.

The last 30 years have seen significant growth in the research on hope theory, demonstrating the applicability and relevance of the theory that has built its own “epic story.” A comprehensive search of the literature that examined the central tenets and correlates of hope theory was conducted across multiple databases, including Ovid (PsycInfo), Web of Science, PubMed, EbscoHost (ERIC and ERC), and Scopus. To explore evidence of the growth and reach of this theory across disciplines and applications, we mapped this literature over time (see Figure 1) and across disciplines (see Figure 2). It should be noted that given the focus on the methodological and meta-theoretical foundations of this manuscript, the review was limited to peer-reviewed journal articles and doctoral theses. It does not represent the significant number of chapters and books that have also been published, nor the articles that have utilized hope theory as a theoretical argument to explain their findings, both of which further evidence the significant impact and application of this theory.

Apart from a slight decline in publications over the 2020/2021 years, which could be explained by the significant impact of the global pandemic on research, there has been a consistent growth pattern in the development of the evidence base for hope theory. Snyder’s theory has also attracted researchers across a broad range of disciplines and applications, which has seen an expansion beyond psychology to a range of applications such as education, sport, psychotherapy, organizational science, and medicine (see Figure 2).

Placing the development of this theory in a sociohistorical context is essential in elucidating some of the dominant ways of knowing or epistemology that underpin the evidence base. Psychological researchers, particularly in the context of building an evidence base to argue for the merits of a new field such as positive psychology, have been grounded in a specific sociohistorical narrative of what constitutes new knowledge (Shotter, 1993). In this context, it is unsurprising that positivist approaches have dominated the assumptions that underpin hope theory development and subsequent research methodologies. They arose in the sociohistorical context of Seligman’s call to arms for a “rigorous” approach to building knowledge of what makes life worth living, driven by the presiding narrative in psychological research at the time that “rigorous” equated to knowledge derived in quantitative terms.

Hope theory was also developed within the context of the rise of motivational literature and cognitive revolution occurring during this period. Snyder (2002) himself comments on the significant influence of Karl Menninger. The latter actively encouraged the prioritization of cognitive processes, with emotions being conceptualized as a secondary affective response to cognitive appraisals of agency and pathways planning. What resulted was a theory of hope as a way of thinking, with emotional responses proposed to be a consequence of the experience of hope rather than a fundamental contributor to driving goal-related performance (Snyder et al., 1999).

The language that Snyder used to communicate the theoretical tenets of this theory indicates a dynamic process, such that adaptive behaviors and characteristics are both the cause and consequence of hope (Snyder, 2002). However, when driven by a process of knowledge acquisition that necessitates the development of measurement scales through factor analysis, the consequence was an operationalization of hope as individual factors rather than an interconnected construct. The resulting literature thus predominantly focuses on the contributory roles of these two factors—both independently and as a combined factor—than on the dynamics between them. Further, the contributions of emotional responses or other types of causal attributions that enable hope to emerge have also been overlooked. While Snyder incorporates the feedback and feedforward loops of emotions and cognitions in his theoretical model, this is not operationalized in his measure of hope. The more reductionistic nature of the methodologies to date also makes it harder to interpret the potential multi-directionality of these relationships and the mechanisms through which hope emerges.

### The Landscape of Hope in University Students

The shortcomings and limitations discussed above are well evidenced in a review of the literature in the university student population. A review of over 60 empirical studies on young people in higher education has highlighted the dominance of positivist epistemological approaches underpinning much of the research. There is no doubt that these methodologies have made a significant contribution to the knowledge that young people with a high sense of hope exhibit adaptive psychological and school-related functioning (see Table 1). For example, this body of evidence has demonstrated that hope is related to numerous factors relevant to the success of young people during their tertiary studies. Hope has direct associations with academic achievement (Snyder et al., 2002; Collins et al., 2009; Davidson et al., 2012; Feldman and Kubota, 2015; Feldman et al., 2015; Griggs and Crawford, 2017), as well as a range of characteristics related to academic success, including perceived control and exam performance (Crane, 2014), coping strategies for study (Onwuegbuzie and Snyder, 2000), persistence (Muwonge et al., 2017), psychological grit (Cavazos Vela et al., 2018), self-efficacy (Davidson et al., 2012; Macaskill and Denovan, 2013), higher engagement and motivation (Dixson, 2019), autonomous learning, social self-efficacy and self-esteem (Macaskill and Denovan, 2013).

**TABLE 1:** Summation of research testing hope theory propositions, correlates, and outcomes in young people in higher education.

Author(s), year	Approach	Methods/Design	Antecedents	Correlates and outcomes	Mediators/Moderators	Location
				*Academic achievement and/or graduation success*		
Snyder et al., 2002	Deductive	Regression (longitudinal)		GPA and Graduation status		United States
Savage and Smith, 2007	Deductive	Regression		Degree attainment		United States
Collins et al., 2009	Deductive	Regression		Assessment performance		United States
Papantoniou et al., 2010	Deductive	Path analysis		Grades	Learning strategies	Greece
Davidson et al., 2012	Deductive	Quasi-experimental		Grades*		Israel
Feldman et al., 2015	Deductive	Quasi-experimental		GPA*		Israel
Feldman and Kubota, 2015	Deductive	Path analysis		GPA	Academic hope** and academic self-efficacy	United States
Griggs and Crawford, 2017	Deductive	Path analysis		GPA	Core self-evaluations	United States
				*Characteristics related to study success*		
Onwuegbuzie and Snyder, 2000	Deductive	Correlational		Coping strategies for study		United States
Davidson et al., 2012	Deductive	Correlational		Sense of coherence, self-efficacy*		Israel
Macaskill and Denovan, 2013	Deductive	Experimental		Autonomous learning, course self-efficacy (agency only) Social self-efficacy Self-esteem		United Kingdom
Muwonge et al., 2017	Inductive	Path analysis		Persistence**		Uganda
Vela et al., 2018	Inductive	Regression		Psychological grit		United States
Dixson, 2019	Deductive	Cluster analysis		Higher engagement and motivation		United States
Luo et al., 2019	Deductive	Path analysis		Learning outcomes (cognitive and non-cognitive)		Taiwan
				*Goal progress/attainment*		
Feldman et al., 2009	Deductive	Path analysis (longitudinal)		Goal attainment	Goal specific hope**	United States
Feldman and Dreher, 2012	Deductive	Experimental		Goal progress		United States
Crane, 2014	Deductive	Regression		Exam performance, approach motivations, perceived control		Australia
Cheavens et al., 2019	Deductive	Regression		Important, prosocial, long-term, and challenging goals		United States
				*Wellbeing and adjustment*		
Chang and DeSimone, 2001	Deductive	Path analysis		Psychological adjustment (direct and indirect)	Appraisals and coping	United States
Denovan and Macaskill, 2013	Inductive	Interpretive/phenomenological		Stress and coping in transition to university		
Liu et al., 2017	Inductive	Regression		Adjustment to collective trauma		United States
Griggs and Crawford, 2017	Deductive	Path analysis		Emotional wellbeing	Core self-evaluations	United States
				*Career choice/vocational calling*		
Phillips, 2011	Deductive	Path analysis		Vocational calling (for women but not men)		United States
Feldman and Dreher, 2012	Deductive	Experimental		Vocational calling and life purpose		United States
Eren, 2015	Inductive	Path analysis		Satisfaction with career choice and sense of responsibility		Turkey
Buyukgoze-Kavas, 2016	Deductive	Regression		Career adaptability and resilience		Turkey
				*Predictors of hope*		
Ahmet and Umran, 2014	Deductive	Regression	Authenticity*			Turkey
Soria and Stubblefield, 2015	Deductive	Regression	Strengths- awareness and self-efficacy			
Cole et al., 2019	Deductive	Path analysis	Gender role conflict (negative relationship)	Gender socialization–conformity to masculine norms (agency only)		United States
Luo et al., 2019	Deductive	Path analysis	Social support, belonging, self-esteem			Taiwan
Chang et al., 2019	Deductive	Path analysis	Positive affect	Life satisfaction	Hope agency but not pathways	China
				*Individual differences*		
Chang and Banks, 2007	Inductive	Regression	Life satisfaction, problem-solving style, positive affect, problem orientation	Variations in levels of agency and pathways thinking between Latino, European, Asian, and African Americans		

*This table is not exhaustive but rather represents a sub-set of the research in this population. It was scoped to include core relationships relevant to adaptive university experience for students.*

*Unless otherwise stated, the relationships in the table are related to trait measures of hope.*

*Related to state measure of hope.

**Related to domain measures of hope.

The literature has evidenced strong links between hope and measures of psychological and emotional wellbeing in higher education students (Chang and Banks, 2007; Wandeler and Bundick, 2011; Macaskill and Denovan, 2013; Griggs and Crawford, 2017; Bernardo et al., 2018; Chang et al., 2019). Therefore, hope is an influential protective factor for mental health and wellbeing, making it particularly valid, valuable, and relevant to the challenging transition periods both into and out of tertiary studies. The buffering and building effects hope provides are relevant for building capability to thrive and managing significant challenges. For example, hope mediates psychological adjustment, even in the face of trauma and adversity (Liu et al., 2017), and acts as a protective factor for suicide risk (Davidson and Wingate, 2011; Lucas et al., 2020).

It is also pertinent that hope may be particularly important for those in lower socioeconomic situations, as it is neither significantly related to intelligence (Snyder et al., 2002) nor income (Gallup, 2009). In general, high hope individuals are energetic and intrinsically motivated; able to set clear goals based on their own standards rather than others, and perceive obstacles as challenges that they can overcome with contingency planning (Chang, 1998; Snyder, 2002; Lopez, 2010; Gallagher et al., 2017). Increased hope can act as an enabling factor for those in lower socioeconomic situations, broadening perspectives on the possibilities available and providing motivation to tap into resources to support goal achievement (Dixson et al., 2017).

Hope may also be considered a robust social leveler. Hope can mediate the relationship between socioeconomic status and academic achievement (Dixson et al., 2017). It is predictive of adaptive functioning in an educational setting, even when controlling for intelligence (Snyder et al., 1997), prior academic history (Gallagher et al., 2017), and self-esteem (Snyder et al., 2002). There is also evidence that hope is particularly important in predicting student resilience, especially in response to uncertainty (Goodman et al., 2017). Indeed, as studies begin to emerge from the recent global pandemic, we can see the role that hope played in navigating some of the extraordinary challenges facing students. For example, a recent study of nearly 6,000 Chinese students in the first wave of lockdowns indicated that hope moderated the relationship between family functioning, loneliness, and mental health (Pan et al., 2021). This research evidences that hope is a critical construct to explore further as we prepare our youth for a volatile, uncertain, complex, and ambiguous (VUCA) world. However, it does not tell us the story of the mechanisms through which these positive associations are achieved. In essence, returning to our Star Wars analogy, this got us straight into the action while acknowledging an untold history still to explore.

### The Missing Pieces of the Story: Identifying Core Gaps in the Research

In recent years, this missing storyline has begun to be addressed in the hope literature. Several studies utilized path analysis to examine the mediation or moderation role that agency and pathways thinking play in the links to adaptive outcomes. For example, Luo et al. (2019) tested a comprehensive model of the role different aspects of hopeful thinking play in linking to various factors that support learning outcomes, such as teacher and peer support, self-esteem, and belonging. However, the extant literature predominantly focused on demonstrating the utility of hope, missing a depth of examination on some of the core tenets of the theory. This includes its antecedents and the pathways through which hope develops. Cheavens et al. (2019) cite this as their core motivation in a recent study that validated that dispositional hope was related to pathways-generating behaviors and goal setting.

A deeper analysis of the hope literature in university students shows that researchers have not yet thoroughly examined whether hopeful cognitions lead to positive affect or indeed whether the emotional experience is a mechanism that develops agency or pathways thinking. Snyder’s theory is that these create an iterative and reciprocal feedback system, but how this has been operationalized and evaluated in the current literature does not build up a “whole picture” of this dynamic process. The two-factor measurement of hope and subsequent analyses of its antecedents, correlates, and outcomes precludes an analysis of the dynamic interaction between these factors to facilitate adaptive relationships, including the mechanisms through which the theoretical reciprocal feedback system functions. Hope also forms a nomological network with other expectancy variables such as self-efficacy, locus of control, and optimism that have been shown to lead to adaptive outcomes (Tennen et al., 2002). While evidence has demonstrated that these variables are distinct but related constructs (Magaletta and Oliver, 1999), much of the research has used path analyses to explain their moderation or mediation impact on adaptive outcomes. However, we believe that this “component” approach to the operationalization of hope does not fully represent the rich and complex interactions that may account for these relationships. With this model of measurement we cannot account for the ways in which pathways thinking and agency thinking may interact in different circumstances, limiting our knowledge and resulting practice to enhance opportunities for hope to emerge.

Despite claims that hope is malleable, there is also limited experimental research in this population that examines how hope develops or whether interventions based on the premises of hope theory effectively increase hope. The small number of experimental studies with young adults have mainly focused on how short hope interventions affect academic performance outcomes (Macaskill and Denovan, 2013; Feldman et al., 2015; Harris, 2015), with some evidence of interventions increasing levels of hope (Davidson et al., 2012; Feldman and Dreher, 2012). Furthermore, a meta-analysis of hope interventions in both community and clinical settings reveal only small effect sizes and inconsistent results depending on the context in which they are delivered (Weis and Speridakos, 2011). Integrated interventions that include other practices based on adaptive constructs such as gratitude or psychological capital (a multi-component construct that includes hope, self-efficacy, resilience, and optimism), have shown increases in hope (Bauman, 2015; Baumsteiger et al., 2019), which further indicates the need to understand the different mechanisms that facilitate the development of hope. Added to this is a lack of inductive or exploratory approaches in research designs that enable a more nuanced picture of hope emerging to be examined. In fact, of all the studies reviewed, only a small handful utilized a mixed-methods or qualitative design, and these were predominantly doctoral theses.

Our review has highlighted that more research is needed to develop a deeper understanding of the factors and interactions that enable hope to emerge in young people. However, we argue that this needs to extend beyond the linear models prevalent in existing meta-theoretical assumptions and methodologies. This is evident from examining the gaps in the evidence base we have discussed thus far and the dearth of hope in our young people. For example, the current landscape reveals that less than half (46%) of Australian and New Zealand school students could be classified as hopeful (Gallup, 2021), indicating that a significant portion of our youth lack abundant ideas and energy for the future. Unfortunately, this trend is replicated in other OECD countries (Gallup, 2018) and highlights a “wicked” problem that is complex, arises from non-linear dynamics, and may have multiple possible causes (Peters, 2017). To address such complexities calls for an expansion in approaches underpinning theoretical and methodological designs, allowing us to examine the complex interactions and factors that impact hope development in young people.

## Adopting a Systems View of Hope

Hope theory, as a cognitive model centered on the individual, is grounded in the liberal individualist sociohistorical context in which it was conceived; however, the emergence of post-positivist and post-modern epistemologies signify growing awareness of the challenges inherent in defining truth in a way that transcends context and abstracts individuals from their environment (Gergen, 1990; Ward et al., 2015; Goldman and O’Connor, 2021). The development of social epistemologies such as social constructionism, that recognize the effects of social interactions and social systems, helps redress this imbalance (Goldman and O’Connor, 2021), and recent developments in positive psychology have acknowledged this with a call toward interdisciplinary perspectives that better address the complexity of human behavior (Kern et al., 2020; Lomas et al., 2020). In an eloquent analogy, Lomas et al. (2020) acknowledge the opportunity in the power and energy generated by the last three decades of research to now propel us forward with the emergence of a new wave in positive psychology; one that moves beyond the individual to embracing complexity. It is with this philosophical intent that we argue that the intersection of systems science and more recent developments in wellbeing science can help address some of the limitations inherent in the current meta-theoretical propositions of hope theory and may inform and energize the next wave of research in this area.

Optimal functioning is recognized to be the outcome of complex and interactive processes, predispositions, and experiences (Roffey, 2015). It includes multiple contributory factors—personal and environmental (Keyes, 2006)—that occur in a nested ecological system. This dynamic ecological system encompasses the individual and many other layers of influence that support and guide development (Bronfenbrenner, 1979). While this approach to understanding optimal functioning is well established in many fields (e.g., sociology, anthropology, biology), positive psychology has been criticized for ignoring the influence of the larger context in favor of its focus on the individual (Kern et al., 2020). However, recent advances have begun to address this limitation in different contexts, such as Lomas et al.’s (2015) proposal of the Layered Integrated Framework Example (LIFE) for applied positive psychology and Williams et al.’s (2016) Inside-Out-Outside-In (IO-OI) model of workplace happiness. However, this criticism is still relevant to Snyder’s hope theory in its acontextual perspective of individual capacity.

Snyder et al. (1991) were quite clear in arguing that they had drawn a clear boundary around the individual, stating that their phenomenological conceptualization of hope could be considered egocentric in that it taps into how an individual *perceives* their ability to move toward goals, with external environmental influences being incorporated only through the lens of how the individual appraises them in relation to agency and pathways thinking. However, more recent advances in the development of hope theory among individuals from more collectivist social settings have expanded this to demonstrate the validity of an external and internal locus of hope. This additional dimension represents the influence of external agents (family, peers, spiritual) in the development of agency thinking (Bernardo, 2010; Du and King, 2013; Bernardo et al., 2018; Bernardo and Mendoza, 2020), providing evidence of the importance of broader system influences in the emergence of hope.

Furthermore, consideration of the dynamics within the human system also provides the opportunity to re-consider the interplay between emotions, cognitions, and somatic experiences that can influence the emergence of hope. One of the limitations of the meta-theoretical assumptions of Snyder’s theory lies in a dualism view that sees the mind as separate from the body and operating independently from the physical world (Buetow, 2007). It is now well-established that mental and physical wellbeing are intimately and bi-directionally linked (Kemp and Quintana, 2013; Steptoe et al., 2015). The vagus nerve, for example, provides a vital structure that communicates between mind and body, providing the opportunity for both thoughts and behavior to influence aspects of wellbeing such as hope (Mead et al., 2021). The solid evidence base that has emerged in recent years regarding the interdependency between various internal factors and the experience of wellbeing calls for a deeper examination of the dynamic interplay of elements within the human system that can contribute to the emergence of hope.

We argue that re-imagining hope theory within a dynamic systems lens can help shine a light on the multiple contributory factors that facilitate the emergence of hope. We aim to expand the theoretical mechanisms that may facilitate Snyder’s conceptualization of a dynamic motivation system that enables goal-directed behavior, reflecting developing knowledge of the interplay *between* the human system and *within* social systems. Our goal in expanding these theoretical tenets is to operationalize some of the dynamics left on the “editing floor” in the methodological translation of hope theory. For example, an extension of Snyder’s two-factor model to a multi-component dynamic systems model can be achieved by including a more contextual motivational component (WhyPower) at the intrapersonal level, and a social-contextual component (WePower) at the interpersonal level. In the following sections, we address these proposed theoretical expansions to hope theory, including the theoretical mechanisms through which these components could contribute to the emergence of hope.

### WhyPower (Intrapersonal Context)

One of the guiding assumptions underpinning hope theory is that humans are goal-oriented (Snyder, 1989, 1995; Snyder et al., 1991). Snyder (2002) positions the importance of goals being of sufficient value to the individual to sustain conscious thought characterizing high hope; however, this criterion is not well represented in the operationalization of hope. One item on the hope trait scale measures an individual’s capacity to “come up with many ways to get the things in life that are important to me” (Snyder et al., 1991). While this is clearly linked to pathways thinking around goals, it does not account for the goal-directed energy that valuable goals can ignite. Incorporating a measure of “sufficient value” in the individual’s goals into a model of hope can provide vital insights into the mechanisms that may facilitate the emergence of hope and may be of particular importance for our research agenda’s population, namely, emerging adulthood. We argue that this is best operationalized as a sense of meaning in our goals or WhyPower.

A search for meaning is a critical developmental task for adolescents (Bronk, 2013; Damon and Malin, 2020). While definitions of meaning vary widely, there is a consensus that meaning has two major components: comprehension and purpose (Steger, 2018a). Comprehension involves making sense and integrating experiences, while the purpose component involves actively pursuing long-term goals that reflect one’s identity but also transcend narrow self-interests (Steger, 2018b). Studies have shown that both these components are positively associated with measures of adaptive functioning in young people (Abe, 2016). In their review of the links between hope and meaning, Feldman et al. (2018) note that the two constructs are “close cousins,” both influencing and contributing to the other, with an average correlation of 0.67. This relationship is also reflected in the links between Reker and Wong’s (1988) conceptualization of meaning; “cognizance of order, coherence and purpose in one’s existence, the pursuit and attainment of worthwhile goals, and an accompanying sense of fulfillment” (p. 221) and key elements of hope theory. Snyder (2002) himself suggested that hope and meaning are “companions,” as he proposed that self-reflective hope thoughts lead to a sense of meaning.

While the strong links between meaning and hope have been demonstrated both longitudinally (e.g., Mascaro and Rosen, 2005) and cross-sectionally (e.g., Feldman and Snyder, 2005), the mechanisms through which this occurs have not yet been established. The comprehension component of meaning may provide valuable insights into how individuals develop a sense of agency and pathway thinking. For example, the process of making sense and integrating experiences may be a mechanism that facilitates a belief in one’s capacity to move toward their goals effectively, as well as expediting divergent thinking that supports the development of pathways planning. In contrast, the purpose component may be a mechanism that facilitates goal-directed energy. Purpose also serves as a self-organizing principle that stimulates goals and manages behavior; it is imperative in guiding decisions about the use of finite resources and likely to lead to greater persistence (McKnight and Kashdan, 2009). Thus, a sense of WhyPower in ones’ goals may lead to enhanced hope through providing energy and motivation with structure and direction (Mascaro and Rosen, 2005).

It is relevant to note that the search for meaning for many young people creates a sense of disconnect rather than leading them to a sense of purpose. One reason may be the context in which this occurs. For example, while many psychologists view purpose from a primarily individualistic perspective (e.g., Ryff, 1989; Damon et al., 2003), Keyes (2011), a sociologist, argues for a conceptualization that reflects that our lives are interwoven within a social construction. He suggests that when viewed through the lens of complete human development, purpose is not just about our own individual sense of direction, but also whether our lives are constructive and contribute to the collective. He terms this authentic purpose, defined as “a quality of being determined to do or achieve an end…that employs one’s gifts, brings a deep sense of worth or value, and provides a significant contribution to the common good” (p. 286).

The role of social connectedness and relationships as a source of meaning has been well documented (e.g., Delle Fave et al., 2011), making it an important component of WhyPower. Furthermore, in a comparison of the differential effects of hope and optimism on various aspects of wellbeing, it was shown that hope was more important in contributing to the more purposeful components of wellbeing (Gallagher and Lopez, 2009). While the inclusion of WhyPower into a dynamic systems model of hope seeks to operationalize some of the cognitive and affective mechanisms at the individual level, it does not fully address the interplay between the individual and their interpersonal context, especially in terms of their access to resources that can facilitate hope.

### WePower (Interpersonal Context)

Bernardo (2010) has argued that a limitation of Snyder’s approach is that it does not consider whether the pathways or sense of agency are self-determined or may involve external agents. Expanding the horizon to examine the multi-directional links between individuals and their social context may be particularly relevant when discussing an adolescent population due to the strong developmental need for social engagement (Siegel, 2014). The developmental stage of late adolescence/emerging adulthood incorporates a second sensitive period of brain maturation that triggers important health behaviors, and studies have demonstrated the important protective factors that social patterns can provide in shaping adolescent wellbeing trajectories (Viner et al., 2012). Therefore, it is proposed that integrating an interpersonal perspective into an expanded model of hope theory may enhance our understanding and facilitate better outcomes for young people in higher education. We operationalize this as a sense of connectedness, or WePower, representing an individual’s ability to tap into resources within their social system.

A fundamental meta-theoretical assumption of Snyder’s hope theory is that hope both acts as a resource *and* facilitates the acquisition of other resources that support healthy development and achievement. Resources can be defined as internal or external entities that are either valued and relevant in their own right, or can be used to obtain valued ends (Hobfoll, 2002). While hope theory has focused on internal resources to date, we propose that external resources are equally important, as are the interrelationships between both forms. For example, how does social connectedness (external) impact the perception of access to resources (internal)? This may be particularly pertinent to students who have moved from being a “big fish in a small pond” in their secondary school context to being “one of many fish in a large pond” in their university context, impacting their perception of access to resources.

Social resources play an essential role in goal attainment and enhanced wellbeing (Hobfoll, 2002), and thus are a critical factor to incorporate in a dynamic systems perspective of hope. Kelly’s (1966) seminal work on the relationships between social resources and wellbeing demonstrated the importance of people’s perception of access to resources within their ecological environment. He described an ecological interplay in which resources are transferred between people and their social settings, demonstrating the importance of expanding the horizon beyond the individual to explore how their experience within an interpersonal context may facilitate the emergence of hope. For example, the sharing of experiences with others may be a crucial factor that builds social bonds, allows individuals to soothe the experience of negative emotions that arise from stressful events, and tap into the knowledge and experience of others to help create pathways toward goals (Smyth et al., 2012). Lee and Gallagher (2018) found that high hope individuals actively seek the support of others in working toward their goals but are also likely to support the goal pursuits of others that serve to strengthen social bonds. Their research highlights the dynamic interplay between the individual and their interpersonal context in developing hope, supporting the need for an expanded perspective.

It is important to note that while these two additional components of WhyPower and WePower have been articulated as separate elements, we argue that hope is best conceptualized as an emergent property that cannot be fully understood by breaking the construct down into its constituent parts. Instead, it is theorized as an energy system derived from the dynamic interplay between the parts. However, how to build up whole pictures of social phenomena poses a significant challenge that has created some controversy amongst systems thinkers as to what the best methodological approach is to achieve this (Flood, 2010).

## What We Measure (And How) Matters

One of the inherent limitations in methodological approaches that arise from a philosophical perspective that takes “an atomistic, ontological view of the world as comprising discrete, observable elements and events that interact in an observable, determined, and regular manner” (Collins, 2010, p. 38) is an oversimplification of dynamic processes. Logical positivist epistemology relies on reducing phenomena to the simplest elements and thus may limit the capacity to analyze the complexity and inherent “messiness” that characterizes human functioning, including reducing influences of the environment.

Given this landscape of knowledge development, it is unsurprising to see quantitative research designs dominating the literature in hope theory to date. Friedman (2003) argues that this seemingly religious devotion to one method and their underlying epistemologies are a flawed form of “methodoltry, the undue elevation of a method to a sacred artifact” (p. 817). While elements of this claim ring true, it is perhaps not a fair representation of the state of play in hope research to date. Indeed, these methods have built robust confidence in the impact of hopeful thinking and its role in adaptive functioning. What is less clear from this literature, driven by deductive reasoning in research design, is the complex interactions that enable hope to emerge. Snyder’s theory was seeded from his conversations with participants in his studies on excuse-making, but he has predominantly taken a top-down approach to theory development rather than a grounded theory approach. There is no doubt that his theoretical propositions draw on a solid evidence base, but this was not grounded in the lived experience of hope. We argue that there is more backstory (a prequel to the trilogy!) that would help articulate some missed nuances inherent in the experience of hope.

Some scholars have sought to remedy this limitation, but this has predominantly focused on more marginalized populations such as domestic violence support workers (Crain and Koehn, 2012) and African American gay men living with HIV (Hergenrather et al., 2013). To our knowledge, there is only one study that has examined the lived experience of hope in university students, centered on prospective teachers and their hopes for their teaching careers. The results indicated hope oriented around an active/passive axis, providing support for some of the goal-oriented cognitive components present in Snyder’s model, as well as a generalized positive expectation that is more representative of the construct of optimism (Eren and Yeşilbursa, 2017). Participants also reported that both internal and external sources, such as peers, family, and friends, contributed to the experience of hope, providing further support for the need to incorporate an interpersonal dimension. These results demonstrate how qualitative studies can complement and enrich an understanding of the lived experience of hope and how it impacts adaptive outcomes, such as teacher retention.

**FIGURE 3:**
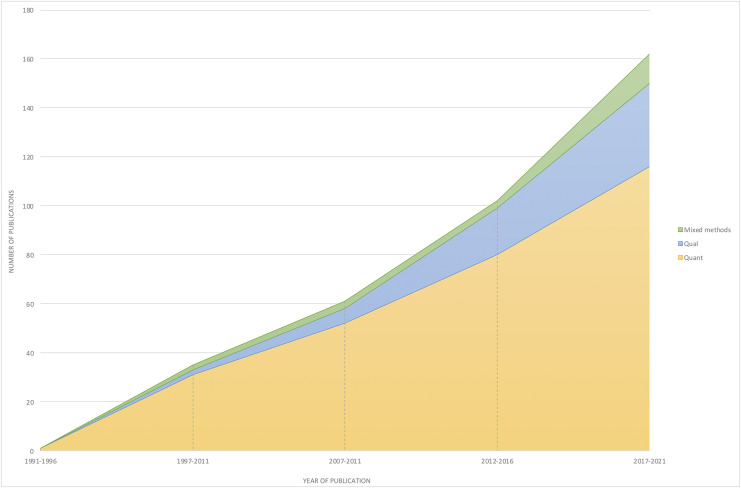
Analysis of the type of research method (quantitative, mixed-methods, qualitative) utilized by year.

Studies of hope in recent years suggest a shift to more mixed-methods and qualitative designs (see Figure 3). However, this still represents a very small proportion of studies (approximately 10% in this review) consistent with trends reported in the broader positive psychology literature (Donaldson et al., 2015). This trend is to be expected in a field that initially embraced a perspective of positivism that views qualitative research as less valuable and scientific (Mruk, 2006). Even when scholars aim to bring more methodological pluralism to their research, they have been thwarted by the dominant narrative of what constitutes scientific knowledge. For example, Shane Lopez, one of the leading scholars in hope research, reported being asked to reduce the qualitative aspects of a mixed-methods paper he submitted to a top positive psychology journal (personal communication, cited in Friedman, 2008).

Expanding methodologies to include more qualitative components can facilitate inquiry into some of the under-researched areas of investigation in hope theory, such as enabling a deepened picture of *how* hope is experienced and the nature of *how* it acts as a protective or enabling factor in adaptive functioning. Qualitative methodologies are ideal for this type of research question because they value the messiness and complexity of the human experience in a sociocultural environment, which can build upon our existing knowledge base. We are not arguing that they are a better approach, but rather that developing methodological diversity increases knowledge formation (Hefferon et al., 2017). Said another way, qualitative approaches can add color and texture to the lines that have been drawn from the quantitative approaches to date. An interdisciplinary approach that integrates more holistic and inductive methods to examine the emergence of hope can help drive the next level of knowledge in the development of hope theory.

## A “New Hope” in Taking an Integrative Perspective

Several scholars have argued that consilience, the principle of bringing together evidence from seemingly independent sources to converge in a common groundwork of explanation, is one of the most promising pathways to scientific advancement (Wilson, 1998; Siegel, 2014; Scharmer, 2016). To achieve this requires a move away from the traditional manner of conducting research that leans toward homogenous and disciplinary silos, toward a more heterogeneous and interdisciplinary approach (Rhoten, 2004). Indeed, broadening both our epistemological and methodological horizons can enable us to reveal new and ground-breaking insights. However, it can also present unique challenges in integrating different ways of knowing; an ongoing challenge in interdisciplinary research, which requires mastery of specific competencies to facilitate respectful dialogue to deepen understanding and integrate different epistemological and methodological approaches (Larson et al., 2011).

Law (2016) underscores the importance of reflective practice in this endeavor, suggesting we “re-search” to integrate different perspectives. Re-searching refers to the practices of engaging in a reflective process throughout the entire life-cycle of research; from ongoing questioning about the assumptions that underpin our research design and selection of methods, through to self-questioning our assumptions and sociocultural context that influences our selection and interpretation of data (Law, 2016). Methodologies that acknowledge the co-creative process of knowledge-making with participants are also essential to achieve this aim (Alcoff, 1991). Unfortunately, methods rooted in positivist epistemologies typically assume the researcher possesses greater interpretative and analytical expertise and knowledge than the research participants (Kleinsasser, 2000).

Shotter (1993) also provides a strong argument for this approach in broadening our knowledge development. He positions the importance of “knowing from within” as a third way of knowing, that complements our traditional focus on “knowing that” and “knowing how” in psychological research. Given the lack of diversity in positive psychology research dominated by Western, Industrialized, Educated, Rich, and Democratic (WEIRD) populations (Rao and Donaldson, 2015), this “knowing from within” can give rise to a more nuanced perspective that is representative and validating of different views. Utilizing more inclusive participatory approaches that give voice and empower the participants themselves in developing knowledge can help bridge some of this gap.

The research agenda we are proposing utilizes a methodology that explicitly facilitates co-creation and participant agency in the research process. Using approaches that enable this, such as Participatory Narrative Inquiry (PNI), can add value in shedding light on participants’ meaning and attributions to make sense of the experience of hope (Czarniawska, 2004). The origins of the PNI methodology are in the systems sciences, influenced by the development of Cynefin, a conceptual framework that helps make sense of influences in a complex and complicated environment (Snowden, 2002). PNI is an approach that senses patterns in the social system through the analysis of stories. The participants play a pivotal role in analyzing those stories to understand the system’s complexity (Kurtz, 2014). Stories have long been used to help us navigate complex social problems, as words are used as tools for meaning-making and constructing mental models (Shotter, 1993; Gottschall, 2012). The inherent simplexity in using stories is that they are both nouns and verbs and thus can provide both research data and methodology for inquiry.

By involving participants in sharing stories and the sense-making process of research, we empower them to give voice to their own experiences and play a key role in contributing to knowledge development. It may also be that this method can provide a two-pronged role as both inquiry and intervention. For example, in a participatory narrative inquiry into mental health recovery, participants described the method as giving them hope, providing them with “a ladder” that facilitated autonomy and direction to climb out of the loneliness and isolation of depression and reconnect with society and work (Torrissen and Stickley, 2018).

Narrative has been described as a two-way reflexive process in which language is used as the vehicle to “construct, to organize, and to attribute meaning to our stories” (Anderson, 1997, p. 213). This process enables the storyteller to make sense of their experience through story sharing and invites the researcher into this sense-making process, giving them unique insight into lay theories of how hope emerges and is experienced. The use of narrative methods rather than surveys also allows us to represent and integrate changes and events in our lives into a comprehensive story. We can include causes and consequences of events, plots, subplots, and overarching themes to weave a coherent narrative of our experience (Smyth et al., 2012). The richness of stories as a source of data and methodology for investigating wicked problems lies in their inherent nature, that is, “stories form complex emergent patterns and all complex patterns have stories” (Kurtz, 2014, p. 633).

Participatory Narrative Inquiry is an excellent example of methodological pluralism that integrates various data sources and ways of knowing (Friedman, 2008). For example, data derived from a more positivist epistemology can be integrated, forming part of the material for sense-making. They provide data into one perspective, while data from methodologies rooted in social constructivist epistemologies offer another view, including a gateway into the mental models that drive these perspectives (Kurtz, 2014). The triangulation of data supports the development of a deeper perspective of the subject of inquiry. PNI can also incorporate analytical methods from various disciplines such as systems mapping, natural language processing, and participatory theater. This provides a unique process for integrating knowledge from different disciplinary perspectives and different meta-theoretical philosophies of knowledge.

Complex social problems deserve diverse perspectives to address the multiplicity and interconnectedness of potential causes and contributory factors. We contend that stories may be an under-tapped resource in the use of interdisciplinary research designs to examine the complexity of the human experience in a way that balances the boundaries of time and resources without compromising cohesiveness.

## Conclusion

Our manuscript has sought to pave the way forward in the next generation of research in hope theory by outlining a “storyboard” that explores both the backstory of the development of hope theory and a roadmap to uncover some of the as yet untold stories of hope. One of our goals in this critical review of hope theory was to pay homage to the scholarly icons of Rick Snyder and Shane Lopez, whose legacies live on in the impact their work has had across a broad range of disciplines. Their choice to “get straight into the action” has paved the way for a significant number of eminent scholars who have followed in their footsteps and taken up the charge to carry on this vital work, providing us with crucial insights into the merits of hope as a fundamental resource to support thriving.

By analyzing the roots of the development of Snyder’s hope theory, we have provided insights into the sociohistorical context that influenced the meta-theoretical assumptions that underpin hope theory. This context helps us understand some of the technical limitations that have impeded the operationalization of hope theory and subsequently led to several gaps in the ensuing research base. Perhaps the most significant of these is the impact of the lost dynamics between elements that facilitate the emergence of hope. Of course, we know the story does not end here, but rather this may provide the necessary “cliff-hanger” that creates the impetus and energy for the subsequent development of this story.

We propose that this is best achieved through a dynamic systems reconceptualization of hope as a pathway to addressing these limitations. This research agenda aims to create a new storyline that expands our operationalization of hope to deepen understanding of the dynamic interactions between the elements that create the unique alchemical reaction of hope. This may include introducing two new “characters,” namely, WhyPower and WePower, expanding the theoretical horizons of hope to integrate systemic intrapersonal and interpersonal perspectives.

However, to translate this vision to action, it is imperative that we broaden our methodological approaches to facilitate an examination of the complexities and interdependencies in such a model. In the same way that we have seen significant advances in technology since the first episodes of Star Wars that have enhanced the cinematic experience and storytelling, we can also draw on more sophisticated interdisciplinary methods now available to advance the study of hope. We advocate a pragmatist approach that pays homage to the wealth of knowledge generated through the methodologies that have dominated the research to date, while intentionally selecting diverse methods to broaden and deepen our understanding of the emergence of hope in young people. Engaging in research covering the full spectrum of epistemological perspectives can enable us to develop richer pictures of positive psychology’s fundamental theories and principles; and, in doing so, realize the vision of “a new hope” for the field.

## Author Contributions

RC conceptualized the research questions for this review and theoretical expansion of Snyder’s theory, developed the search protocols, conducted the research using PRISMA-Sc, developed and organized the database, including coding 80% of the data, and wrote the manuscript. PW and LO added contextual insights and provided contributions in shaping arguments. JC-M coded studies by discipline and performed the mapping analysis. PW and RC contributed to the manuscript revision, read, and approved the submitted version.

## Conflict of Interest

The authors declare that the research was conducted in the absence of any commercial or financial relationships that could be construed as a potential conflict of interest.

## Publisher’s Note

All claims expressed in this article are solely those of the authors and do not necessarily represent those of their affiliated organizations, or those of the publisher, the editors and the reviewers. Any product that may be evaluated in this article, or claim that may be made by its manufacturer, is not guaranteed or endorsed by the publisher.
