# Corrigendum: “A new hope” for positive psychology: a dynamic systems reconceptualization of hope theory

**DOI:** 10.3389/fpsyg.2023.1292756

**Published:** 2023-10-06

**Authors:** Rachel Colla, Paige Williams, Lindsay G. Oades, Jesus Camacho-Morles

**Affiliations:** Centre for Wellbeing Science, Melbourne Graduate School of Education, University of Melbourne, Parkville, VIC, Australia

**Keywords:** systems dynamics, interdisciplinary, hope theory, methodology, meta-theoretical

In the published article, there was an error in Figures 1, 3. These figures were published in the wrong order. That is, Figure 1, correctly captioned, showed the image of Figure 3 and vice versa. The corrected Figures 1, 3 appear below.

**Figure 1:**
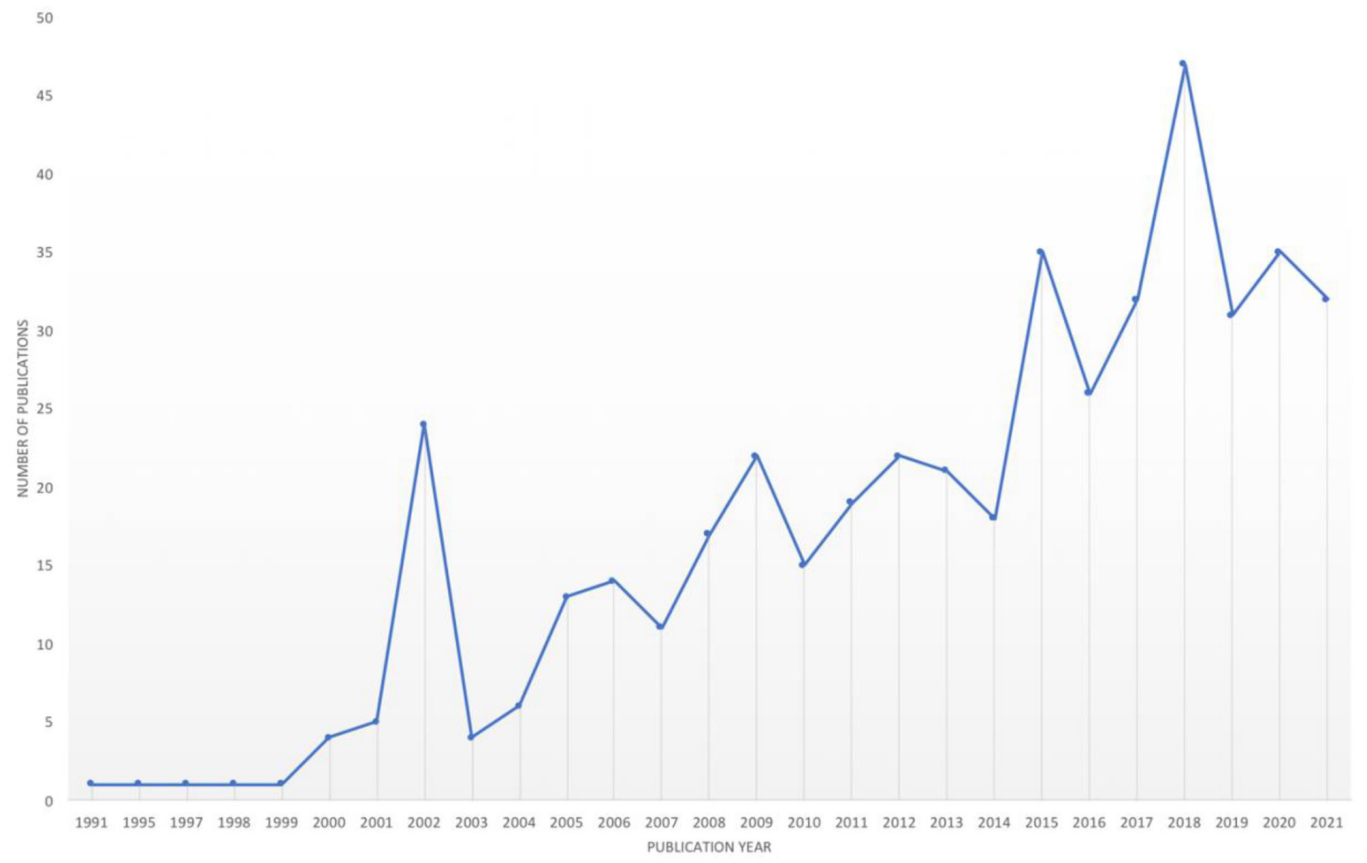
Growth in number of peer-reviewed publications in hope theory by year (1991–2021).

**Figure 3:**
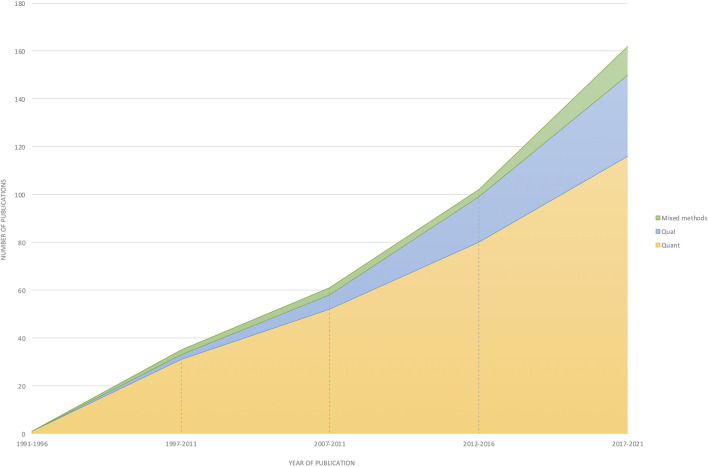
Analysis of the type of research method (quantitative, mixed-methods, qualitative) utilized by year.

The authors apologize for this error and state that this does not change the scientific conclusions of the article in any way. The original article has been updated.

